# Evaluating the Impact of Benign Prostatic Hyperplasia Surgical Treatments on Sexual Health

**DOI:** 10.3390/biomedicines12010110

**Published:** 2024-01-05

**Authors:** Aris Kaltsas, Zisis Kratiras, Athanasios Zachariou, Fotios Dimitriadis, Nikolaos Sofikitis, Michael Chrisofos

**Affiliations:** 1 Third Department of Urology, Attikon University Hospital, School of Medicine, National and Kapodistrian University of Athens, 12462 Athens, Greece; ares-kaltsas@hotmail.com (A.K.); zkratiras@gmail.com (Z.K.); 2 Department of Urology, Faculty of Medicine, School of Health Sciences, University of Ioannina, 45110 Ioannina, Greece; azachariou@uoi.gr (A.Z.); nsofikit@uoi.gr (N.S.); 3 Department of Urology, Faculty of Medicine, School of Health Sciences, Aristotle University of Thessaloniki, 54124 Thessaloniki, Greece; difotios@auth.gr

**Keywords:** BPH, surgery, sexuality, TUR-P, HoLEP, ThuLEP, Rezum

## Abstract

Benign prostatic hyperplasia (BPH), a prevalent condition in older men, is often managed through various surgical interventions. This narrative review aims to explore the impact of these surgical treatments on sexual function, a critical aspect of patient quality of life often overlooked in BPH management. The methodology encompassed a thorough review of contemporary surgical techniques for BPH, including prostate resection, enucleation, vaporization, and minimally invasive therapies such as UroLift, Rezum, and Aquablation. Additionally, the focus was on patient-centered outcomes, with a special emphasis on sexual health following surgery. Findings reveal that, while surgical interventions effectively alleviate BPH symptoms, they often have significant repercussions in sexual function, including erectile and ejaculatory dysfunction. However, emerging techniques demonstrate potential in preserving sexual function, underscoring the need for patient-centric treatment approaches. The study highlights the complex interplay between BPH surgery and sexual health, with minimally invasive treatments showing promise in balancing symptom relief and sexual function preservation. In conclusion, the study advocates for an integrated, interdisciplinary approach to BPH treatment, emphasizing the importance of considering sexual health in therapeutic decision-making. This narrative review suggests a paradigm shift towards minimally invasive techniques could optimize patient outcomes, marrying symptom relief with quality-of-life considerations. The need for further research in this domain is evident, particularly in understanding long-term sexual health outcomes following different surgical interventions for BPH.

## 1. Introduction

Benign prostatic hyperplasia (BPH) is a prevalent medical condition characterized by the non-malignant enlargement of the prostate gland [1]. A condition that is notably common among males aged 50 years and above, its prevalence rises significantly with age. Studies estimate that around 50% of men in their 50s are diagnosed with BPH, and this proportion escalates to 80% for those aged 80 and above [2]. This disorder is known to induce lower urinary tract symptoms (LUTS) which include, but are not limited to, increased urinary frequency, urgency, diminished urine flow, and nocturia [3,4]. Given its widespread occurrence and clinical implications, BPH is not merely a health concern but a broader public health issue that necessitates ongoing attention [5,6]. 

In recent decades, even with substantial advancements in medical treatments for BPH, surgical intervention retains a pivotal position in comprehensive patient care. Such surgical treatments are especially recommended for patients resistant to or intolerant of medicinal interventions, or those experiencing complications attributed to BPH [7]. The principal aim of surgical procedures is to ameliorate LUTS by excising or diminishing the obstructive prostatic tissue, thereby enhancing the overall quality of life (QoL) [8]. Various surgical techniques, such as transurethral resection of the prostate (TURP), enucleation of the prostate, and prostate arterial embolization, are employed based on the severity of symptoms and patient characteristics [9,10,11].

A concurrent and paramount concern in the surgical management of BPH is its potential repercussions in sexual function. The gravity of this concern is underscored by multiple studies indicating potential associations between surgical interventions for BPH and various sexual dysfunctions, such as erectile dysfunction (ED), ejaculatory disturbances, and changes in libido [12,13]. Nonetheless, there is a subset of research suggesting neutral or even beneficial impacts of BPH surgeries on sexual function [14]. Moreover, the intersectionality of BPH treatments with other medical conditions, such as overactive bladder or even mood disorders, can exacerbate these side effects, culminating in a compounded decline in the patient’s holistic well-being [15]. Given this, an interdisciplinary approach that integrates considerations of sexual function within the purview of BPH surgical management is indispensable. Decisions surrounding surgical strategies and adjunctive medical treatments must be underpinned by a nuanced understanding of their implications for sexual health [16,17,18].

This manuscript is committed to furnishing a rigorous assessment of the interplay between surgical modalities for BPH and their concomitant effects on sexual function. By juxtaposing various surgical techniques against their respective sexual outcomes, we aim to underscore the clinical significance of BPH and advocate for a patient-centric approach in therapeutic decision-making.

## 2. Pathophysiology of BPH Related to Sexual Function

### 2.1. Exploring the Pathophysiology of BPH

BPH is a multifaceted condition whose exact cause remains unclear. However, several key factors are widely recognized as playing a significant role in its development.

Hormonal Imbalance: The balance between androgens and estrogens is critical for prostate health. Androgens, particularly testosterone and dihydrotestosterone (DHT), are essential for normal prostate function [19,20]. Elevated estrogen levels or diminished androgen activity have been linked to prostatic tissue enlargement. This enlargement results from hormonal interactions that stimulate growth in both the epithelial and stromal cells of the prostate [19,20].

Chronic Inflammation: Inflammation within the prostate gland is a major factor in BPH’s development. Inflammatory molecules such as cytokines and chemokines can promote cellular growth and remodeling [21]. Notably, an increase in interleukin-8 (IL-8) has been observed in BPH tissues [21]. Additionally, inflammation may contribute to LUTS, which are commonly associated with BPH [22].

Metabolic Factors: Obesity and insulin resistance are increasingly implicated in BPH. Obesity increases intra-abdominal pressure, alters hormone levels, and promotes inflammation and oxidative stress, all of which are risk factors for BPH [23]. Insulin resistance, a characteristic of metabolic syndrome, is also linked to prostatic tissue growth, possibly through the action of insulin-like growth factors (IGFs) [24,25].

Vascular Changes: Vascular dynamics play a significant role in BPH. Increased angiogenesis, the formation of new blood vessels, has been observed in BPH tissues. Changes in vascular structure and function may contribute to symptoms related to bladder outlet obstruction and LUTS [26].

Genetic and Epigenetic Influences: Genetic predisposition is a key factor in BPH development. Specific genetic variants have been linked to an increased risk and severity of BPH [27]. Epigenetic changes, such as DNA methylation and histone modifications, can influence gene expression and contribute to the progression of BPH [27].

In summary, BPH is a complex condition influenced by hormonal imbalances, chronic inflammation, metabolic factors, vascular changes, and genetic/epigenetic factors. Understanding these interrelated mechanisms is vital for developing effective treatments and personalized management strategies for BPH.

### 2.2. BPH and Sexual Function: A Complex Relationship

#### 2.2.1. BPH’s Impact on Sexual Health

BPH can influence sexual function through various mechanisms that involve both physiological and psychological elements. A thorough understanding of these mechanisms is crucial for grasping the impact of BPH on sexual function and for formulating effective treatment strategies.

One of the ways BPH can affect sexual performance is by physically obstructing the prostatic urethra. McVary et al. suggest that prostate gland hypertrophy in BPH can lead to bladder outlet blockage [28]. This obstruction may directly contribute to difficulties in achieving ejaculation, and could lead to ED due to disrupted semen flow and impaired penile blood flow. Such obstruction can alter the normal flow of semen during ejaculation, resulting in retrograde ejaculation (RE) or a decreased force of ejaculation [29]. Additionally, a physical barrier might impair normal blood flow to the penis, further exacerbating ED [30].

Another factor is BPH’s impact on hormonal balance. BPH is associated with altered levels of androgens and estrogens, leading to elevated estrogen levels and decreased androgen sensitivity. These hormonal shifts can specifically disrupt physiological mechanisms related to sexual function, potentially leading to sexual dysfunctions such as decreased libido and ED [29]. Elevated estrogen levels, for instance, may interfere with the nitric oxide/cyclic guanosine monophosphate (NO/cGMP) pathway, which is essential for penile erection and can thus affect erectile function [30].

A role of inflammation in linking BPH to sexual dysfunction has been proposed. Chronic inflammation in the prostate, commonly observed in BPH, can release inflammatory mediators affecting adjacent tissues’ function. Mirone et al. indicate that these inflammatory cytokines and chemokines can disrupt physiological processes related to sexual function, potentially contributing to conditions like ED and reduced sexual desire [30]. This inflammation may also be involved in fibrosis and tissue remodeling, which can further impact sexual function by altering the structural integrity of the prostate and surrounding tissues [30].

The medications used to treat BPH, such as alpha-blockers and 5-alpha reductase inhibitors (5ARIs), can also influence sexual function. While alpha-blockers can relax the prostate and bladder neck’s smooth muscles to enhance urine flow, they might also negatively affect ejaculation, leading to issues such as reduced ejaculatory volume or anejaculation [31]. On the other hand, 5ARIs have been linked to sexual side effects like ED and decreased libido, directly impacting patients’ sexual experiences and satisfaction [29]. 

Moreover, psychological factors can also influence the relationship between BPH and sexual function. Symptoms related to BPH, such as frequent urination and nocturia, can lead to anxiety and stress, which can adversely affect sexual desire and performance, thereby impacting a patient’s overall sexual health and quality of life [32]. The effects of BPH on sexual function can also reduce sexual satisfaction and strain relationships [30]. Addressing these psychological aspects is essential when evaluating and managing sexual dysfunction in BPH patients.

In conclusion, BPH can impact sexual function through multiple avenues, including physical obstruction, hormonal changes, inflammation, medication side effects, and psychological factors. For healthcare practitioners, understanding these interconnected pathways is vital to provide comprehensive and effective treatment for patients with BPH and accompanying sexual dysfunction. By acknowledging these aspects, clinicians can offer more effective treatments to improve sexual function and overall quality of life for those diagnosed with BPH.

#### 2.2.2. Post-Surgical Sexual Health Challenges

**Figure 1:**
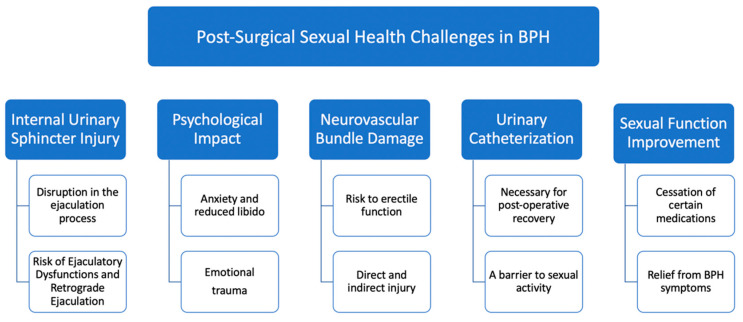
Exploring the impact of BPH surgery on sexual health.

The surgical intervention for BPH often brings significant relief from urinary symptoms, yet it can also inadvertently impact sexual function. This worsening of sexual health post-surgery is attributed to a variety of mechanisms, each interplaying in a complex manner [33,34,35,36,37,38]. To better understand these complexities, Figure 1 visually summarizes the key mechanisms through which surgical intervention for BPH may affect sexual function.

Injury to the internal urinary sphincter: The internal urinary sphincter, situated within the genitourinary tract, plays a pivotal role in the ejaculation process. This involuntary smooth muscle is responsible for controlling the flow of semen in the proper antegrade direction during ejaculation [39]. However, BPH surgeries can sometimes inadvertently damage this sphincter. As a consequence, many patients report ejaculatory dysfunctions (EjD), with RE being a common outcome. In this condition, semen flows backward into the bladder instead of being ejected out of the penis [40]. The disruption or injury to the internal urethral sphincter is the chief cause of this retrograde flow, highlighting the importance of safeguarding this muscle during surgical interventions [41].

Psychological impact of the surgery: The surgical treatment for BPH does not just have physiological ramifications; it also exerts a considerable psychological toll on the patient. The very realization of having undergone surgery in such an intimate area can manifest in anxiety, leading to diminished sexual desire and heightened dissatisfaction. These psychological aftereffects can be compounded by previous relationship experiences that might have left emotional scars. Past traumas, unresolved emotional conflicts, and the pressure to perform sexually post-surgery can all coalesce, potentially precipitating or exacerbating ED. Addressing this facet is paramount, as the mind’s health is closely intertwined with physical well-being [42,43,44].

Neurovascular bundle damage: One of the more intricate aspects of BPH surgery relates to the proximity of the neurovascular bundles. These bundles, crucial for erectile function, are delicate structures that can be inadvertently harmed during the surgical procedure. Direct injury might happen if there’s a puncture to the capsule surrounding the prostate. Even though such events are infrequent, their occurrence can have long-term repercussions for erectile function. On the other hand, indirect injuries, often from heat sources used in surgery, present another risk. The debate continues about the exact mechanisms and extent of heat-induced damage, but it underscores the importance of surgical precision and care [34,45].

Urinary catheterization and its impediments: The postoperative period following BPH surgery necessitates certain medical interventions to aid recovery, one of which is urinary catheterization. This involves placing a tube into the bladder to facilitate urine drainage. While medically essential, the presence of a catheter can be a psychological and physical barrier to sexual activity. The discomfort and the very presence of a foreign body can deter patients from engaging in sexual intercourse, at least in the immediate recovery phase [46].

Despite these challenges, it is noteworthy that some patients experience an improvement in their sexual function following BPH surgery. This paradoxical outcome can arise from several factors, such as the discontinuation of medications affecting sexual health and the relief from the bothersome urinary symptoms of BPH.

#### 2.2.3. Improvement in Sexual Function Post-Surgery

Following surgical treatment for BPH, some patients experience an unexpected yet welcome improvement in their sexual function. This positive change typically arises from two primary factors.

The role of BPH medication: One of the notable aspects that intertwine BPH with sexual function is the medication regime patients are often prescribed. Before undergoing surgery, many patients are on a variety of drugs intended to manage their symptoms. Each of these drugs, while beneficial in managing BPH, can come with side effects that impinge on sexual performance [31]. When these medications are discontinued post-surgery, some men notice a marked improvement in their sexual function, highlighting an important aspect of postoperative care. However, this raises ethical questions about the balance between managing BPH symptoms and preserving sexual health.

Alleviation of LUTS: The relationship between LUTS and sexual dysfunction is complex. As many aging men will attest, the two conditions often manifest concurrently [47]. Improvements in LUTS post-surgery can lead to enhanced sexual function. Nonetheless, the decision to opt for surgery also involves ethical considerations. Healthcare providers must weigh the potential benefits of surgical intervention against the risks, including possible impacts on sexual function. This requires a patient-centered approach, ensuring informed consent and considering individual patient preferences and overall health status. Moreover, the ethical implications extend to the choice of surgical methods. Different procedures may have varying impacts on sexual function, and patients must be fully informed about these outcomes to make decisions that align with their values and quality of life goals.

In essence, the journey of understanding sexual function post-BPH-surgery is a mosaic of physiological, pharmacological, and psychological elements. As the medical community continues to refine surgical techniques and enhance patient care protocols, considering these ethical dimensions becomes paramount in providing holistic and patient-centered care.

## 3. Impact of BPH Surgery on Sexual Function

### 3.1. Understanding Sexual Dysfunction: Insights and Implications in BPH Treatment

The majority of research studies tend to prioritize the examination of surgical and functional outcomes associated with BPH surgery, while the investigation of sexual consequences is often neglected or insufficiently explored. Despite the high prevalence of BPH in the elderly population, a significant proportion of men delay seeking medical consultation until they encounter troublesome urinary symptoms [48]. 

The functionality of male sexual processes is a multifaceted interaction involving aspects such as psychological, neurogenic, vascular, and hormonal elements. While encompassing various aspects including sexual desire, erectile function, orgasmic function, ejaculatory function, and sexual pleasure, it is common for only erectile and ejaculatory functions to be assessed. EjD and ED are the most frequently reported sexual difficulties. Although α-blockers or 5ARIs are often prescribed as the first-line treatment for men with LUTS, surgical intervention becomes a viable alternative for patients experiencing severe LUTS or complications associated with BPH, such as acute urinary retention [49,50]. Research has shown that surgical interventions for LUTS provide prompt relief in mitigating symptoms associated with BPH. However, these surgical therapies are more likely to induce sexual dysfunction compared to other therapeutic approaches. 

The International Index of Erectile Function (IIEF) and its abbreviated versions, namely IIEF-5 and IIEF-EF, are extensively employed as validated questionnaires in assessing erectile function. Similarly, the Male Sexual Health Questionnaire (MSHQ), along with its specialized variant focusing on EjD, known as the Male Sexual Health Questionnaire—Ejaculatory Dysfunction Short Form (MSHQ-EjD-SF), are extensively used tools for assessing ejaculatory function [51,52]. It is important for clinicians to acknowledge that many patients with BPH commonly experience preoperative sexual dysfunction, including both ED and EjD [53]. EjD encompasses a range of symptoms related to ejaculation and orgasm, including RE, premature ejaculation, delayed ejaculation, anejaculation, painful ejaculation, and reduced strength, volume, or pleasure associated with ejaculation [54]. The examination of orgasmic function, sexual pleasure, and sexual desire is seldom explored in research, and the use of validated tools to assess these outcomes is rare. In recent decades, there has been a growing emergence of innovative surgical interventions for BPH. These interventions aim to achieve comparable results in relieving LUTS while simultaneously limiting the occurrence of sexual adverse effects. Technologies such as Aquablation, prostatic artery embolization (PAE), UroLift (Pleasanton, CA, USA), and Rezum (Maple Grove, MN, USA) have shown encouraging outcomes in terms of preserving sexual function [55]. In conclusion, most studies pertaining to BPH surgery that evaluate sexual outcomes consist of case series without a control group, a methodological limitation that restricts the robustness of the data obtained [41,56]. To further elucidate these surgical options, the following Table 1 provides a detailed overview of the various prostate surgery techniques employed in BPH treatment.

### 3.2. Prostate Resection Techniques: Evaluating Sexual Health Outcomes 

The gold standard surgical therapy for BPH in individuals with prostate sizes ranging from 30–80 mL is TURP. This surgery is widely recognized as the reference method in the majority of comparative research [57]. The correlation between EjD and TURP has been well documented in recent medical literature, highlighting the significance of TURP as a preferred surgical treatment for BPH [58,59]. Studies indicate an incidence rate of 62–75% for EjD, especially RE, post-TURP [60,61,62]. Moreover, Taher et al. discovered a 13–14% chance of developing ED after TURP, with a higher likelihood in those with lower preoperative nocturnal penile tumescence [61,63]. 

In assessing the impacts of M-TURP and B-TURP on sexual function, studies have employed tools like the erectile function component of the IIEF-ED and the ejaculatory domain of the male sexual-health questionnaire (Ej-MSHQ) [51,52]. These studies revealed comparable effects of both M-TURP and B-TURP on erectile and ejaculatory functions. The IIEF-15 was used to compare impacts on general sexual function, including erection, orgasmic function, sexual desire, intercourse satisfaction, and overall satisfaction. No significant differences were observed between M-TURP and B-TURP over a twelve-month follow-up period [52,64]. Additionally, a comprehensive meta-analysis corroborated these findings, showing similar levels of erectile function, as measured by the IIEF-5, at twelve months post-procedure for both M-TURP and B-TURP [65]. Interestingly, no significant correlation was found between the volume of resected prostate and the increased risk of sexual dysfunction [52,64,66,67]. Patients who have undergone brachytherapy for prostate cancer are also more likely to experience urinary incontinence post-TURP [68,69]. In transurethral incision of the prostate (TUIP), there is a reduced incidence of EjD. TUIP has a lower propensity, compared to some other treatments for BPH, in inducing RE [70].

### 3.3. Prostate Enucleation Methods: Assessing Impacts on Sexual Function

Holmium laser enucleation of the prostate (HoLEP) has emerged as a significant therapeutic method for BPH, especially in patients with prostates larger than 100 g [71]. It has been compared favorably to open prostatectomy in terms of effectiveness, offering benefits like less blood loss and shorter hospital stays [72]. However, it also carries inherent risks, including a 21% incidence of painful ejaculation and a 70% occurrence of RE over a six-month period post-HoLEP, as noted by Meng et al. [73]. A rise in occurrences of early morning erections was seen in 15% of patients post-HoLEP [74]. 

Briganti et al. compared HoLEP with the established gold standard TURP, revealing no significant differences in postoperative erectile function and rates of RE and reduced ejaculate volume between the two treatments [74]. However, a high prevalence of RE (76.6%), reduced ejaculatory volume (18.3%), and painful ejaculation (3.3%) was documented after one year of follow-up post-HoLEP. While a marginal improvement in IIEF erectile function scores was observed, a notable decline in orgasmic function was also reported, potentially due to increased RE and decreased ejaculatory function [74]. Zong et al. concluded that there were no significant disparities in sexual dysfunction between HoLEP and TURP [75]. This distinction in findings underscores the complexity of sexual function outcomes post-surgery and highlights the importance of considering different dimensions of sexual health in evaluating treatment impacts. 

A systematic review and meta-analysis, including seven randomized controlled trials (RCTs), examined the comparative effectiveness of HoLEP and TUR-P. The findings indicated that short- and mid-term IIEF-5 scores were similar between the two procedures. However, in the long term, HoLEP demonstrated considerably superior outcomes in terms of IIEF-5 scores [76]. Additionally, two more meta-analyses found no significant difference in rates of mid-term RE [77]. The effects on erectile function and RE are similar in both the HoLEP and TURP procedures [74,78]. There was no observed loss in erectile function from the initial measurements in any of the groups. Additionally, it was shown that approximately 75% of sexually active patients experienced RE after the HoLEP procedure. The available data indicate that ejaculation and the feeling of orgasm are the two domains most affected by HoLEP [35]. The success rate of preserving ejaculatory function with HoLEP has been reported to be as high as 46.2% in some patient populations [79]. 

While HoLEP demonstrates certain advantages and risks, it is also crucial to consider the impact of other surgical methods, such as open prostatectomy (OP), in the context of sexual function. In evaluating the impact of OP on sexual function, specifically through the lens of the IIEF-5 scores, two meta-analyses provide insightful data [80,81]. These studies compared the overall safety and outcomes of OP, performed via a transvesical approach, with other procedures such as bipolar transurethral enucleation of the prostate (B-TUEP) and HoLEP. Notably, the IIEF-5 scores, a measure used to gauge erectile function, showed no significant differences between OP and the other procedures at various follow-up intervals, including three, six, twelve, and twenty-four months. This suggests that, despite the longer catheterization and hospitalization times, and the higher incidence of blood transfusions associated with OP, its impact on erectile function, as measured by IIEF-5 scores, is comparable to that of B-TUEP and HoLEP [80,81]. A RCT conducted to compare the outcomes of thulium laser enucleation of the prostate (ThuLEP) and B-TURP revealed a statistically significant disparity in IIEF-5 scores, with ThuLEP demonstrating superior results after twelve months [82].

### 3.4. Prostate Vaporization Procedures: Exploring Effects on Sexual Health

The GreenLight laser vaporization of the prostate (photoselective vaporization of the prostate [PVP]) has been identified as a viable alternative treatment to TURP for managing BPH. This laser therapy facilitates the rapid vaporization of the transitional zone of the prostate [58,83]. The GOLIATH prospective study’s findings indicate no statistically significant differences in rates of RE and IIEF-5 scores between PVP and TURP over 1-year and 2-year follow-up periods, respectively [84,85]. New onset RE incidence rates were found to range from 30% to 67.1%, with an additional 5.4% risk of painful ejaculation post-PVP [36,84,86]. While single-institution studies have shown no adverse effects of PVP on erectile function [36,87], a recent meta-analysis by Li et al. revealed that, out of nine BPH treatments examined, PVP was the only procedure to negatively impact short-term postoperative erectile function [78]. It is noteworthy that studies using higher levels of laser energy have a greater likelihood of resulting in sexual dysfunction [88]. 

A systematic review and meta-analysis of five RCTs was conducted to compare the efficacy of all three types of ‘GreenLight’ lasers with TURP in terms of RE rates. The study revealed no significant differences in RE rates across the different treatment modalities [77]. Furthermore, numerous other studies have also documented similar findings, indicating no discernible difference in erectile function outcomes between OP/TURP and PVP [89,90]. Nevertheless, the scores on the IIEF-5 showed a significant decline at six, twelve, and twenty-four months among patients who had a pre-operative IIEF-5 score higher than nineteen [91].

As we continue to explore various vaporization techniques for prostate treatment, attention must also be given to emerging methods that are currently under investigation. Among these is the diode laser vaporization technique. Recent studies investigating this method have specifically focused on its implications for late complications, such as ED. The findings indicate that diode laser vaporization, in terms of late complications like ED, demonstrates no significant differences compared to other prostate treatment methods. This suggests that the impact of diode laser vaporization on sexual function is minimal, making it a noteworthy consideration in the array of prostate treatment options [92].

### 3.5. Alternative Ablative Techniques in BPH Treatment

#### 3.5.1. Rezum: Evaluating Sexual Function Post Convective Water Vapor Energy Ablation

Building on the insights into Convective Water Vapor Energy Ablation, commonly known as Rezum, and its innovative approach using radiofrequency-generated thermal energy, we now turn to an in-depth study of its clinical outcomes. The Rezum system, by employing a unique mechanism of action that involves water vapor to effect cell necrosis, has shown promise in treating LUTS while preserving sexual function. The following discussion delves into the findings of comprehensive research and trials assessing the long-term effectiveness and safety of Rezum in treating BPH-related symptoms and its impact on sexual health [93,94,95].

McVary et al. conducted a comprehensive prospective cohort study, the broadest and most extensive of its kind, involving 136 patients tracked over up to 4 years [94]. The study found a risk of anejaculation below 3% post-operation, which resolved within three months. A reduction in ejaculatory volume was observed in 2.9% of cases, decreasing to 1.5% during the same period [95,96]. Sexual function was assessed using the IIEF and the MSHQ-EjD over 2 years, showing consistent and stable results. Additionally, the MSHQ-EjD-Bother score, measuring distress associated with EjD, improved substantially over 3 years [97]. Similar findings were reported in other prospective trials by Darson and Dixon, where no significant changes in IIEF and MSHQ-EjD scores were observed post-operation, and no new instances of ED were identified [98,99]. Additional retrospective and crossover investigations have indicated a 3–6% probability of reduced ejaculatory volume, sometimes referred to as REj [93,100]. 

However, it is essential to acknowledge the limitations and specific patient indications for the use of Rezum. The system is intended to relieve symptoms and obstructions, and reduce prostate tissue associated with BPH, and is indicated for men ≥ 50 years of age with a prostate volume of 30 mL to 80 mL, including treatment of the prostate with hyperplasia of the central zone and/or a median lobe. It is not recommended for patients with a urinary sphincter implant, those who have a penile prosthesis, or those with an active urinary tract infection [101,102]. It is crucial to recognize Rezum as a viable, safe, and effective minimally invasive option for patients with urinary retention due to BPH, notably those who are frail with comorbidities and unable to undergo general anesthesia. This treatment provides a therapeutic alternative, particularly for catheterized patients, offering efficient relief with minimal intervention [103].

Incorporating these indications and contraindications helps in counseling patients and stressing the impact on sexual function. More research is required to accurately determine the prevalence of sexual dysfunction, as current data suggest it is seldom documented. Overall, the Rezum system demonstrates long-lasting and prompt alleviation of LUTS with limited and transient effects on sexual function. Further research is needed to compare Rezum’s effectiveness in treating males with BPH and LUTS against existing gold standard treatments.

#### 3.5.2. Aquablation: Advanced Technology and Sexual Health Implications

Aquablation, a groundbreaking treatment for BPH, has demonstrated its potential in addressing LUTS through innovative technology. Utilizing the AquaBeam system, this approach applies high-velocity waterjets under real-time ultrasound guidance, offering a minimally invasive alternative to traditional methods [104]. To enhance understanding of this novel technique, it is crucial to discuss both its indications and limitations. Aquablation has been shown to be as effective as TURP, both subjectively and objectively, for patients with moderate-to-severe LUTS and a prostate volume of 30–80 mL [105,106,107]. However, it remains under investigation to further understand its long-term effects, efficacy, and the optimal methods for post-treatment hemostasis. Patients should be informed about the risk of bleeding and the absence of extensive long-term follow-up data.

The longest prospective trial to date, conducted by Gilling et al., has yielded encouraging findings about sexual outcomes after the treatment, observed over a one-year period. In addition to documenting substantial improvement in LUTS and observing similar occurrences of post-procedural urine urgency and frequency as compared to TURP, the authors also demonstrated the maintenance of ejaculatory function in all patients after the intervention. Patients who underwent Aquablation saw improvements in their IIEF ratings. However, these improvements were not statistically significant, except in the subdomain related to happiness with intercourse [108,109]. 

In the cohort of sexually active males, the incidence of ejaculation was shown to be significantly lower in those who underwent Aquablation treatment compared to those who received TURP, with rates of 10% versus 36%, respectively. No adverse effects related to the surgery were reported during a six-month period [107]. Additionally, the incidence rates of REj were also quite low for Aquablation, with rates of 6% at 3 months, decreasing further to 0.9% by 6 months. Furthermore, the IIEF and MSHQ-EjD scores exhibited no significant changes in the group of men who received the Aquablation procedure, in contrast to the group of men who experienced a decline in scores after TURP [105,107]. The preservation of antegrade ejaculation was found to be somewhat lower in the WATER II study, with a rate of 81%, compared to the 90% rate observed in the smaller prostates of the WATER I study [110]. In the WATER II study, there was also a reported 2% rate of de novo incontinence after twelve months [111]. To date, there have been no documented cases of ED associated with Aquablation treatment [105,107].

#### 3.5.3. Prostatic Artery Embolization: PAE’s Impact on Sexual Function

Building upon the understanding of PAE as a minimally invasive technique with positive impacts on reducing prostate volume and improving LUTS, the focus now shifts to its effects on sexual health [112,113]. While PAE has shown promising results, it is important to note that this technique remains under ongoing investigation to fully ascertain its long-term effects and optimal application. Various studies, including comprehensive meta-analyses and large-scale trials, have delved into the consequences of PAE for parameters like the IIEF. These investigations provide crucial insights into the sexual function outcomes for patients undergoing PAE [114,115].

Multiple investigations have reported an increase in IIEF scores post-procedure in individuals with varying prostate sizes, with no documented instances of newly occurring ED [116,117,118,119]. Additionally, a meta-analysis by Wang et al. revealed noteworthy enhancements in IIEF ratings at the 6- and 12-month post-treatment assessments for individuals who underwent PAE [120]. The latest UK-ROPE trial, conducted prospectively across multiple centers, aimed to evaluate the safety and effectiveness of PAE. The study’s findings further supported the notion that PAE does not have a detrimental impact on sexual function. It also indicated a rate of re-intervention (REj) at 24.1%, which was less than half of that seen in the TURP cohort [121].

Regarding sexual adverse events, the meta-analysis found no statistically significant variations in the mean differences in International Index of IIEF-5 scores between TURP and PAE [11]. A subsequent meta-analysis, including two RCTs, revealed no significant variation in rates of RE [77]. The assessment of erectile function after surgery, as evaluated by IIEF-5, demonstrated a statistically significant improvement in patients who underwent PAE. The average difference in the change of IIEF-5 scores between pre-operative and post-operative measurements was found to be 2.56 points in favor of PAE. In another meta-analysis, PAE was consistently shown to exhibit a reduced prevalence of sexual dysfunction compared to TURP, with an odds ratio of 0.24 [122].

However, it is essential to highlight that PAE is generally less effective than TURP at improving symptoms and urodynamic parameters such as flow rate [123]. The procedural time for PAE tends to be longer than that for TURP, but factors like blood loss, catheterization, and hospitalization time are more favorable with PAE [11]. PAE should be offered to men with moderate-to-severe LUTS who are considering minimally invasive treatment options and are willing to accept less optimal outcomes compared to TURP [124]. It is critical that PAE is performed in facilities where both urologists and trained interventional radiologists collaborate closely for the identification of suitable candidates for PAE [124].

### 3.6. Non-Ablative Techniques for BPH: Preserving Sexual Function

#### 3.6.1. Prostatic Urethral Lift: Long-Term Sexual Function Outcomes 

Building upon the understanding of Prostatic Urethral Lift (PUL), which utilizes the UroLift system and suture-based implants to relieve prostatic obstruction, as a minimally invasive treatment for BPH, we now explore its clinical outcomes. Extensive research has provided valuable insights into PUL’s effectiveness in symptom relief and its impact on sexual function. The following section delves into detailed findings from these studies, highlighting the long-term efficacy and safety of PUL in managing LUTS while preserving sexual health [125,126,127].

Research conducted by Roehrborn et al. on the LIFT cohort presents the most extensive collection of prospectively gathered PUL data. The study’s findings indicate no statistically significant alterations in the scores of the MSHQ and IIEF questionnaires compared to participants’ initial baseline measurements during yearly follow-up assessments. Symptom alleviation related to LUTS was seen within two weeks post-treatment, with preservation of erectile and ejaculatory functioning observed for up to five years post-PUL. Additionally, the study noted limited negative urinary symptoms associated with the procedure, predominantly occurring during the first three months post-operation. These symptoms included dysuria, experienced by 9% of participants, and urge incontinence, experienced by 3% of participants [128].

A separate investigation by McVary et al. indicated notable enhancements in ejaculatory function, specifically a 4% increase in the ability to ejaculate, a 23% improvement in ejaculation intensity, and a 22% rise in ejaculate volume [129]. In a RCT comparing PUL to TURP, it was demonstrated that PUL led to a higher grade of recovery and better preservation of ejaculatory function. No significant changes were observed in either treatment group with respect to ejaculatory performance and bother ratings [130]. A systematic review and meta-analysis revealed that sexual function, specifically in terms of erectile and ejaculatory function, either remained stable or showed slight improvement over a 24-month follow-up period [131,132,133]. Thus, the existing body of research unequivocally supports the assertion that this tissue-conserving method yields moderate and expeditious alleviation of LUTS while safeguarding sexual function. 

However, it is crucial to address that, while PUL enhances the International Prostate Symptom Score (IPSS), peak urinary flow rate (Qmax), and QoL, these improvements are generally less significant compared to TURP at 24 months [134]. PUL is recognized for its low incidence of sexual side effects, yet patients should be informed about the relatively unexplored long-term effects, including the potential for retreatment [135]. PUL is suggested for men with LUTS who prioritize preserving ejaculatory function and have prostates smaller than 70 mL without a middle lobe [136]. This recommendation aims to guide patients in making informed decisions by understanding both the benefits and limitations of PUL, thereby enhancing patient counseling, and emphasizing the impact on sexual function.

#### 3.6.2. Intra-Prostatic Injections and iTIND: Innovations in Minimizing Sexual Dysfunction

Intra-prostatic injections employ various compounds administered directly into the prostate gland to ameliorate LUTS. Among these, Botulinum toxin-A (BoNT-A), fexapotide triflutate (NX-1207), and PRX302 are notable. BoNT-A primarily functions by suppressing neurotransmitter release from cholinergic neurons [137]. The specific mechanisms of NX-1207 and PRX302, both injectable medications, are not fully understood, but experimental evidence suggests that they may induce apoptosis, leading to prostate shrinkage [137]. PRX302, in particular, is a protein with pore-forming properties that is selectively activated by prostate-specific antigen (PSA) and administered via injection to induce apoptosis in prostate tissue. In a cohort study involving 92 individuals, PRX302 showed no sexual side effects over a 12-month follow-up period [138].

Clinical trials, however, have not demonstrated any clinical advantage of BoNT-A over a placebo in managing LUTS due to Benign Prostatic Obstruction (BPO). Conversely, clinical trials have shown NX-1207 to provide clinical benefits over placebo for managing LUTS attributable to BPO. Consequently, intraprostatic BoNT-A injection treatment is not recommended for patients with male LUTS [139].

Additionally, the temporary implantable nitinol device (iTIND) is a medical device functioning like a stent, designed to create incisions within the prostate gland. These incisions aim to remodel both the urethra and the bladder neck, thereby mitigating bladder outlet obstruction. In a study with 19 patients, no instances of EjD were recorded over a 3-year follow-up period [140,141]. There have been no recent reports of ejaculatory dysfunction or ED [142,143]. 

Additionally, it is important to note that ongoing RCTs are actively comparing the iTIND with standard reference techniques. This evolving research is vital for patients and healthcare providers to assess the most up-to-date and effective treatment options, thoroughly weighing the prospective benefits and drawbacks of these novel approaches. Such information is critical in guiding well-informed patient counseling and in comprehensively understanding the potential implications on sexual function.

## 4. Strategies to Preserve Sexual Function Post-BPH-Surgery

Preserving sexual function post-BPH-surgery is crucial for maintaining the overall quality of life for patients. It is essential to assess and discuss sexual function with the patient before deciding on the management strategy for LUTS associated with BPH [144]. Novel minimally invasive treatment alternatives have demonstrated the ability to preserve postoperative sexual function to a better degree, while providing significant relief of LUTS in an equally safe and efficacious manner [41]. Patient perspectives on BPH surgery emphasize the importance of preserving erectile and ejaculatory functions, highlighting the need to consider sexual health in the treatment decision-making process [16].

In order to maintain ejaculatory function, adjustments to surgical procedures have been documented in existing literature [145]. Saman et al. presented a modified approach to PVP, focusing on preserving certain anatomical structures such as the bladder neck muscle fibers, precollicular tissue, and paracollicular prostate tissue. In their study, only 13% of the 160 patients experienced anejaculation. The majority, including 56% of the participants, reported normal ejaculation, while 31% noted a reduction in ejaculation [146]. Alloussi et al. modified the TURP procedure and observed that 91% of their study participants sustained anterograde ejaculation, with these effects lasting up to five years [147]. Kim et al. introduced an alternative approach in HoLEP, preserving the paracollicular and supracollicular tissue. However, their study, which compared this novel approach with the usual HoLEP technique, did not show significant improvement in ejaculatory function. The authors suggested that retaining a greater amount of apical tissue might be crucial for maintaining ejaculation [79]. The conventional simple prostatectomy has evolved with the advent of robot-assisted procedures. A small-scale study by Wang et al. showed that only 1 out of 14 patients experienced RE following this surgery [148]. Although recent advancements have enhanced the potential of conventional procedures to yield favorable sexual outcomes, more research is needed to substantiate these observations.

It is essential to engage in comprehensive discussions with patients about the possible impact of procedures on sexual function. This will enable patients to prepare adequately for any potential decline in sexual function, thereby avoiding unexpected outcomes. Although over 90% of practitioners discuss the potential occurrence of ED related to TURP and PVP, only about 60% actively discuss ED concerns [149]. Albaugh et al. conducted research involving a cohort of 27 individuals diagnosed with prostate cancer and treated with either surgery or radiation. The participants reported experiencing sexual dysfunction within five years after their medical intervention. Most individuals expressed a need for greater awareness of potential adverse sexual outcomes post-operation, as this knowledge would have facilitated a more comprehensive understanding of the probable outcomes. Some patients felt that their healthcare professionals were overly optimistic about post-treatment sexual function. The majority of those affected expressed a need for more support from healthcare practitioners in addressing their sexual issues. Additionally, a significant number of these individuals emphasized the importance of a robust social support network in aiding their recovery. While this research focused on patients diagnosed with prostate cancer, some underlying concepts may be extrapolated to patients who have undergone surgery for BPH [150].

Furthermore, comprehensive discussions with patients about newer, less invasive treatment options as alternatives to TURP, PVP, or HoLEP are important. Although TURP has traditionally been the most effective therapy for BPH, many urologists now prefer other methods, largely due to innovative techniques that offer comparable surgical outcomes to TURP while significantly reducing sexual side effects. Patients should be informed about the availability of these operations to make a well-informed choice regarding their treatment. While there may be limitations on certain procedures in some areas or higher associated costs, a collaborative decision-making approach is recommended to select the most suitable method aligned with the patient’s preferences and objectives. Healthcare practitioners should stay informed about the latest BPH treatments and maintain a comprehensive directory of providers offering alternative treatments.

There is also a noticeable shift among urologists away from TURP, as indicated by recent trends. A study in Australia in 2019, analyzing Australian Medicare data, showed that TURP accounted for 96% of surgical procedures between 1998 and 2008, dropping to 73% between 2008 and 2014. From 2014 to 2017, the study reported that 15.5% of the procedures were PVP, while 7.7% and 5.9% were UroLift and HoLEP, respectively [151]. A similar decrease in TURP rates was observed in the United States, with a significant reduction of 47.6% between 2000 and 2008, attributed to the growing preference for alternative procedures like PVP, transurethral needle ablation (TUNA), and transurethral microwave thermotherapy (TUMT) [152].

## 5. Future Directions and Research Opportunities

Emerging surgical techniques for the treatment of BPH present an opportunity to investigate their potential impact on sexual function. Minimally invasive therapies such as UroLift, Rezum, and Aquablation have garnered interest due to their potential to achieve symptomatic relief while maintaining sexual function [41]. Additionally, procedures like ThuLEP and PAE have shown promise in preserving sexual function post-surgery [153,154]. However, there is a need for further research to comprehensively evaluate the effects of these emerging techniques on sexual outcomes. Comparative studies, such as evaluating the impact of HoLEP on erectile function, can provide valuable insights into the effects of these procedures on sexual function [155,156].

Furthermore, potential areas for research to improve sexual outcomes post-BPH-surgery include investigating the long-term effects of surgical interventions on sexual function. Prospective studies assessing the trajectory of sexual function following BPH surgeries, such as HoLEP, can provide valuable insights into the recovery and maintenance of sexual function over time [157]. Additionally, there is a need for large cohort studies to evaluate the sexual and functional outcomes of patients undergoing emerging procedures like ejaculation-sparing thulium laser enucleation of the prostate (ES-ThuLEP) [158]. Long-term follow-up studies can offer comprehensive insights into the sustained impact of these surgical interventions on sexual function. 

Moreover, research focusing on the comparative effectiveness of prolonged medical therapy versus early surgical treatment for BPH can provide valuable evidence to guide treatment decisions and improve long-term sexual outcomes [159]. Randomized clinical trials comparing the outcomes of these approaches can help determine the most beneficial strategy for preserving sexual function in the context of an aging population. Additionally, evaluating the impact of BPH surgeries on sexual function in conjunction with other chronic illnesses can provide a comprehensive understanding of the complex interplay between various health conditions and sexual function [160].

In conclusion, future research should focus on systematically evaluating the impact of emerging surgical techniques on sexual function post-BPH-surgery. Prospective studies, comparative effectiveness research, and long-term follow-up studies are essential to advance our understanding of the effects of these interventions on sexual outcomes and to identify strategies to improve sexual function in BPH patients.

## 6. Conclusions

The study of surgical interventions for BPH and their effects on sexual function is vital for comprehensive patient care, extending beyond symptom relief. Preserving sexual function post-BPH-surgery is crucial for quality of life and is a fundamental aspect of therapeutic decision-making. This necessitates a nuanced approach that prioritizes patient perspectives on sexual health, alongside the management of LUTS associated with BPH.

Emerging surgical techniques such as UroLift, Rezum, Aquablation, ThuLEP, and PAE have shown promise in balancing symptomatic relief with the preservation of sexual function. These minimally invasive therapies represent a new horizon in BPH treatment, focusing on sexual health as a key patient outcome.

Ongoing research is essential to evaluate the long-term effects of these emerging surgical interventions on sexual function. Prospective studies and large cohort studies, particularly those evaluating innovative procedures like ES-ThuLEP, are vital for understanding the sustained impact on sexual health. Additionally, comparative research on medical therapy versus early surgical intervention is crucial for guiding treatment decisions that prioritize sexual function preservation, especially in older patients. 

Understanding how BPH surgeries intersect with other chronic conditions will also lead to a more holistic approach to care, acknowledging the complex relationship between health conditions and sexual function. In summary, the future of BPH management hinges on systematically evaluating the surgical impact on sexual function post-surgery and incorporating patient-centered care where sexual health is a primary outcome. With continued advancements and robust research, there is potential to significantly improve sexual outcomes for patients undergoing BPH surgery, ultimately enhancing their overall quality of life and well-being.

## Figures and Tables

**Table 1:** Overview of prostate surgery techniques for BPH treatment.

**Prostate Surgery Categories**	** Specific Surgical Procedures **
Resection of the prostate	Monopolar and bipolar transurethral resection of the prostate Holmium laser resection of the prostate Thulium:yttrium–aluminum–garnet laser vaporesection of the prostate Transurethral incision of the prostate
Enucleation of the prostate	Open prostatectomy Bipolar transurethral enucleation of the prostate Holmium laser enucleation of the prostate Thulium:yttrium–aluminum–garnet laser enucleation of the prostate Diode laser enucleation of the prostate Enucleation techniques under investigation: – Minimal invasive simple prostatectomy – 532 nm (‘GreenLight’) laser enucleation of the prostate
Vaporization of the prostate	Bipolar transurethral vaporization of the prostate 532 nm (‘GreenLight’) laser vaporization of the prostate Vaporization techniques under investigation Diode laser vaporization of the prostate
Alternative ablative techniques	Aquablation—image guided robotic waterjet ablation: AquaBeam Prostatic artery embolization Alternative ablative techniques under investigation: – Convective water vapor energy (WAVE) ablation: the Rezum system
Non-ablative techniques	Prostatic urethral lift Intra-prostatic injections Non-ablative techniques under investigation: – (i) TIND

## References

[1] Langan R.C. (2019). Benign Prostatic Hyperplasia. Prim. Care.

[2] Patel N.D., Parsons J.K. (2014). Epidemiology and etiology of benign prostatic hyperplasia and bladder outlet obstruction. Indian J. Urol..

[3] Manov J.J., Mohan P.P., Kava B., Bhatia S. (2020). Benign Prostatic Hyperplasia: A Brief Overview of Pathogenesis, Diagnosis, and Current State of Therapy. Tech. Vasc. Interv. Radiol..

[4] Lloyd G.L., Marks J.M., Ricke W.A. (2019). Benign Prostatic Hyperplasia and Lower Urinary Tract Symptoms: What Is the Role and Significance of Inflammation?. Curr. Urol. Rep..

[5] Zattoni F., Ficarra V., Novara G. (2017). Risk stratification for benign prostatic hyperplasia. Urologia.

[6] Miernik A., Gratzke C. (2020). Current Treatment for Benign Prostatic Hyperplasia. Dtsch. Arztebl. Int..

[7] Devlin C.M., Simms M.S., Maitland N.J. (2021). Benign prostatic hyperplasia—What do we know?. BJU Int..

[8] Alcaraz A., Carballido-Rodriguez J., Unda-Urzaiz M., Medina-Lopez R., Ruiz-Cerda J.L., Rodriguez-Rubio F., Garcia-Rojo D., Brenes-Bermudez F.J., Cozar-Olmo J.M., Baena-Gonzalez V. (2016). Quality of life in patients with lower urinary tract symptoms associated with BPH: Change over time in real-life practice according to treatment—The QUALIPROST study. Int. Urol. Nephrol..

[9] Aho T., Armitage J., Kastner C. (2020). Anatomical endoscopic enucleation of the prostate: The next gold standard? Yes!. Andrologia.

[10] Monreal R., Robles C., Sanchez-Casado M., Ciampi J.J., Lopez-Guerrero M., Ruiz-Salmeron R.J., Lanciego C. (2020). Embolisation of prostate arteries in benign prostatic hyperplasia in non-surgical patients. Radiologia.

[11] Knight G.M., Talwar A., Salem R., Mouli S. (2021). Systematic Review and Meta-analysis Comparing Prostatic Artery Embolization to Gold-Standard Transurethral Resection of the Prostate for Benign Prostatic Hyperplasia. Cardiovasc. Intervent. Radiol..

[12] Becher E.F., McVary K.T. (2014). Surgical Procedures for BPH/LUTS: Impact on Male Sexual Health. Sex Med. Rev..

[13] Borchert A., Leavitt D.A. (2018). A Review of Male Sexual Health and Dysfunction Following Surgical Treatment for Benign Prostatic Hyperplasia and Lower Urinary Tract Symptoms. Curr. Urol. Rep..

[14] Soans J., Vazirian-Zadeh M., Kum F., Dhariwal R., Breish M.O., Singh S., Mahmalji W., Katmawi-Sabbagh S. (2020). Can surgical treatment for benign prostatic hyperplasia improve sexual function? A systematic review. Aging Male.

[15] Eapen R.S., Radomski S.B. (2016). Review of the epidemiology of overactive bladder. Res. Rep. Urol..

[16] Bouhadana D., Nguyen D.D., Zorn K.C., Elterman D.S., Bhojani N. (2020). Patient Perspectives on Benign Prostatic Hyperplasia Surgery: A Focus on Sexual Health. J. Sex Med..

[17] Lepor H. (2016). Alpha-blockers for the Treatment of Benign Prostatic Hyperplasia. Urol. Clin. North Am..

[18] Kim E.H., Brockman J.A., Andriole G.L. (2018). The use of 5-alpha reductase inhibitors in the treatment of benign prostatic hyperplasia. Asian J. Urol..

[19] Nicholson T.M., Ricke W.A. (2011). Androgens and estrogens in benign prostatic hyperplasia: Past, present and future. Differentiation.

[20] Ho C.K., Habib F.K. (2011). Estrogen and androgen signaling in the pathogenesis of BPH. Nat. Rev. Urol..

[21] Fibbi B., Penna G., Morelli A., Adorini L., Maggi M. (2010). Chronic inflammation in the pathogenesis of benign prostatic hyperplasia. Int. J. Androl..

[22] Gandaglia G., Briganti A., Gontero P., Mondaini N., Novara G., Salonia A., Sciarra A., Montorsi F. (2013). The role of chronic prostatic inflammation in the pathogenesis and progression of benign prostatic hyperplasia (BPH). BJU Int..

[23] Parikesit D., Mochtar C.A., Umbas R., Hamid A.R. (2016). The impact of obesity towards prostate diseases. Prostate Int..

[24] Zhao S., Wang Y., Wu W., Yang S., Feng L., Tao F., Ge W., Shen M., Xu W. (2021). Nonalcoholic fatty liver disease and risk of prostatic diseases: Roles of insulin resistance. Andrologia.

[25] Kopp W. (2018). Diet-Induced Hyperinsulinemia as a Key Factor in the Etiology of Both Benign Prostatic Hyperplasia and Essential Hypertension?. Nutr. Metab Insights.

[26] Levin R., Chichester P., Levin S., Buttyan R. (2004). Role of angiogenesis in bladder response to partial outlet obstruction. Scand J. Urol. Nephrol. Suppl..

[27] van Rij S., Gilling P. (2015). Recent advances in treatment for Benign Prostatic Hyperplasia. F1000Res.

[28] McVary K.T., Chughtai B., Miller L.E., Bhattacharyya S.K., Dornbier R.A., Elterman D.S. (2021). Putting Patients Ahead by Leaving Nothing Behind: An Emerging Treatment Paradigm in Minimally Invasive Surgical Therapy for Benign Prostatic Hyperplasia. Med. Devices.

[29] Corona G., Tirabassi G., Santi D., Maseroli E., Gacci M., Dicuio M., Sforza A., Mannucci E., Maggi M. (2017). Sexual dysfunction in subjects treated with inhibitors of 5alpha-reductase for benign prostatic hyperplasia: A comprehensive review and meta-analysis. Andrology.

[30] Mirone V., Sessa A., Giuliano F., Berges R., Kirby M., Moncada I. (2011). Current benign prostatic hyperplasia treatment: Impact on sexual function and management of related sexual adverse events. Int. J. Clin. Pract..

[31] Gacci M., Ficarra V., Sebastianelli A., Corona G., Serni S., Shariat S.F., Maggi M., Zattoni F., Carini M., Novara G. (2014). Impact of medical treatments for male lower urinary tract symptoms due to benign prostatic hyperplasia on ejaculatory function: A systematic review and meta-analysis. J. Sex Med..

[32] Rosen R.C., Giuliano F., Carson C.C. (2005). Sexual dysfunction and lower urinary tract symptoms (LUTS) associated with benign prostatic hyperplasia (BPH). Eur. Urol..

[33] Otani T. (2019). Clinical review of ejaculatory dysfunction. Reprod. Med. Biol..

[34] Muntener M., Aellig S., Kuettel R., Gehrlach C., Sulser T., Strebel R.T. (2007). Sexual function after transurethral resection of the prostate (TURP): Results of an independent prospective multicentre assessment of outcome. Eur. Urol..

[35] Elshal A.M., El-Assmy A., Mekkawy R., Taha D.E., El-Nahas A.R., Laymon M., El-Kappany H., Ibrahiem E.H. (2017). Prospective controlled assessment of men’s sexual function changes following Holmium laser enucleation of the prostate for treatment of benign prostate hyperplasia. Int. Urol. Nephrol..

[36] Spaliviero M., Strom K.H., Gu X., Araki M., Culkin D.J., Wong C. (2010). Does Greenlight HPS() laser photoselective vaporization prostatectomy affect sexual function?. J. Endourol..

[37] Poulakis V., Ferakis N., Witzsch U., de Vries R., Becht E. (2006). Erectile dysfunction after transurethral prostatectomy for lower urinary tract symptoms: Results from a center with over 500 patients. Asian J. Androl..

[38] Park J., Cho S.Y., Cho M.C., Jeong H., Son H. (2017). Changes in Erectile Function after Photoselective Vaporization of the Prostate with a 120-W GreenLight High-Performance System Laser: 2-Year Follow-Up. World J. Mens Health.

[39] Clement P., Giuliano F. (2016). Physiology and Pharmacology of Ejaculation. Basic Clin. Pharmacol. Toxicol..

[40] Bearelly P., Avellino G.J. (2021). The role of benign prostatic hyperplasia treatments in ejaculatory dysfunction. Fertil. Steril..

[41] Leong J.Y., Patel A.S., Ramasamy R. (2019). Minimizing Sexual Dysfunction in BPH Surgery. Curr. Sex Health Rep..

[42] Zhu D., Gao J., Dou X., Peng D., Zhang Y., Zhang X. (2021). Incidence and Risk Factors of Post-Operative Depression in Patients Undergoing Transurethral Resection of Prostate for Benign Prostatic Hyperplasia. Int. J. Gen. Med..

[43] Wang S.C., Chien W.C., Chung C.H., Tzeng N.S., Liu Y.P. (2021). Posttraumatic stress disorder and the risk of erectile dysfunction: A nationwide cohort study in Taiwan: PTSD and erectile dysfunction. Ann. Gen. Psychiatry.

[44] Pavone C., Abbadessa D., Scaduto G., Caruana G., Scalici Gesolfo C., Fontana D., Vaccarella L. (2015). Sexual dysfunctions after transurethral resection of the prostate (TURP): Evidence from a retrospective study on 264 patients. Arch. Ital. Urol. Androl..

[45] Al Demour S.H., Abuhamad M., Santarisi A.N., Al-Zubi M., Al-Rawashdah S.F., Halalsheh O., Carbone A., Pastore A.L., Ahmad M.M. (2022). The Effect of Transurethral Resection of the Prostate on Erectile and Ejaculatory Functions in Patients with Benign Prostatic Hyperplasia. Urol. Int..

[46] Chapple A., Prinjha S., Salisbury H. (2014). How users of indwelling urinary catheters talk about sex and sexuality: A qualitative study. Br. J. Gen. Pract..

[47] McVary K. (2006). Lower urinary tract symptoms and sexual dysfunction: Epidemiology and pathophysiology. BJU Int..

[48] Isa N.M.M., Aziz A.F.A. (2020). Lower Urinary Tract Symptoms: Prevalence and Factors Associated with Help-Seeking in Male Primary Care Attendees. Korean J. Fam. Med..

[49] Lekas A.G., Lazaris A.C., Chrisofos M., Papatsoris A.G., Lappas D., Patsouris E., Deliveliotis C. (2006). Finasteride effects on hypoxia and angiogenetic markers in benign prostatic hyperplasia. Urology.

[50] Simaioforidis V., Papatsoris A.G., Chrisofos M., Chrisafis M., Koritsiadis S., Deliveliotis C. (2011). Tamsulosin versus transurethral resection of the prostate: Effect on nocturia as a result of benign prostatic hyperplasia. Int. J. Urol..

[51] Akman T., Binbay M., Tekinarslan E., Tepeler A., Akcay M., Ozgor F., Ugurlu M., Muslumanoglu A. (2013). Effects of bipolar and monopolar transurethral resection of the prostate on urinary and erectile function: A prospective randomized comparative study. BJU Int..

[52] El-Assmy A., ElShal A.M., Mekkawy R., El-Kappany H., Ibrahiem E.H.I. (2018). Erectile and ejaculatory functions changes following bipolar versus monopolar transurethral resection of the prostate: A prospective randomized study. Int. Urol. Nephrol..

[53] Vickers A.J., Tin A.L., Singh K., Dunn R.L., Mulhall J. (2020). Updating the International Index of Erectile Function: Evaluation of a Large Clinical Data Set. J. Sex Med..

[54] Catania J.A., Oakley L.P., Rosen R., Pollack L.M. (2013). Effects of interview mode on assessments of erectile and ejaculatory dysfunction among men with benign prostatic hyperplasia (BPH). J. Sex Res..

[55] Das A.K., Han T.M., Uhr A., Roehrborn C.G. (2020). Benign prostatic hyperplasia: An update on minimally invasive therapy including Aquablation. Can. J. Urol..

[56] Blachman-Braun R., Ory J., Shah H.N., Ramasamy R. (2021). Is Sexual Function Impacted After Minimally Invasive Surgery for Benign Prostatic Obstruction?. Eur. Urol..

[57] Murad L., Bouhadana D., Nguyen D.D., Chughtai B., Zorn K.C., Bhojani N., Elterman D.S. (2023). Treating LUTS in Men with Benign Prostatic Obstruction: A Review Article. Drugs Aging.

[58] Foster H.E., Barry M.J., Dahm P., Gandhi M.C., Kaplan S.A., Kohler T.S., Lerner L.B., Lightner D.J., Parsons J.K., Roehrborn C.G. (2018). Surgical Management of Lower Urinary Tract Symptoms Attributed to Benign Prostatic Hyperplasia: AUA Guideline. J. Urol..

[59] Deliveliotis C., Liakouras C., Delis A., Skolarikos A., Varkarakis J., Protogerou V. (2004). Prostate operations: Long-term effects on sexual and urinary function and quality of life. Comparison with an age-matched control population. Urol. Res..

[60] Marra G., Sturch P., Oderda M., Tabatabaei S., Muir G., Gontero P. (2016). Systematic review of lower urinary tract symptoms/benign prostatic hyperplasia surgical treatments on men’s ejaculatory function: Time for a bespoke approach?. Int. J. Urol..

[61] Mebust W.K., Holtgrewe H.L., Cockett A.T., Peters P.C. (2002). Transurethral prostatectomy: Immediate and postoperative complications—A cooperative study of 13 participating institutions evaluating 3885 patients. 1989. J. Urol..

[62] Donovan J.L., Peters T.J., Neal D.E., Brookes S.T., Gujral S., Chacko K.N., Wright M., Kennedy L.G., Abrams P. (2000). A randomized trial comparing transurethral resection of the prostate, laser therapy and conservative treatment of men with symptoms associated with benign prostatic enlargement: The CLasP study. J. Urol..

[63] Taher A. (2004). Erectile dysfunction after transurethral resection of the prostate: Incidence and risk factors. World J. Urol..

[64] Mamoulakis C., Skolarikos A., Schulze M., Scoffone C.M., Rassweiler J.J., Alivizatos G., Scarpa R.M., de la Rosette J.J. (2013). Bipolar vs monopolar transurethral resection of the prostate: Evaluation of the impact on overall sexual function in an international randomized controlled trial setting. BJU Int..

[65] Alexander C.E., Scullion M.M., Omar M.I., Yuan Y., Mamoulakis C., N’Dow J.M., Chen C., Lam T.B. (2019). Bipolar versus monopolar transurethral resection of the prostate for lower urinary tract symptoms secondary to benign prostatic obstruction. Cochrane Database Syst. Rev..

[66] Chen Q., Zhang L., Fan Q.L., Zhou J., Peng Y.B., Wang Z. (2010). Bipolar transurethral resection in saline vs traditional monopolar resection of the prostate: Results of a randomized trial with a 2-year follow-up. BJU Int..

[67] Frieben R.W., Lin H.C., Hinh P.P., Berardinelli F., Canfield S.E., Wang R. (2010). The impact of minimally invasive surgeries for the treatment of symptomatic benign prostatic hyperplasia on male sexual function: A systematic review. Asian J. Androl..

[68] Keehn A., Fram E., Garg M., Maria P. (2017). UroLift in Place of Fiducial Markers for Patients With Benign Prostatic Hyperplasia Undergoing External Beam Radiation Therapy. Urology.

[69] Mock S., Leapman M., Stock R.G., Hall S.J., Stone N.N. (2013). Risk of urinary incontinence following post-brachytherapy transurethral resection of the prostate and correlation with clinical and treatment parameters. J. Urol..

[70] Bansal A., Sankhwar S., Kumar M., Jhanwar A., Purkait B., Aeron R., Goel S. (2016). Holmium Laser vs Monopolar Electrocautery Bladder Neck Incision for Prostates Less Than 30 Grams: A Prospective Randomized Trial. Urology.

[71] Gilling P.J., Kennett K., Das A.K., Thompson D., Fraundorfer M.R. (1998). Holmium laser enucleation of the prostate (HoLEP) combined with transurethral tissue morcellation: An update on the early clinical experience. J. Endourol..

[72] Kuntz R.M., Lehrich K., Ahyai S.A. (2008). Holmium laser enucleation of the prostate versus open prostatectomy for prostates greater than 100 grams: 5-year follow-up results of a randomised clinical trial. Eur. Urol..

[73] Meng F., Gao B., Fu Q., Chen J., Liu Y., Shi B., Xu Z. (2007). Change of sexual function in patients before and after Ho:YAG laser enucleation of the prostate. J. Androl..

[74] Briganti A., Naspro R., Gallina A., Salonia A., Vavassori I., Hurle R., Scattoni E., Rigatti P., Montorsi F. (2006). Impact on sexual function of holmium laser enucleation versus transurethral resection of the prostate: Results of a prospective, 2-center, randomized trial. J. Urol..

[75] Zong H.T., Peng X.X., Yang C.C., Zhang Y. (2012). The impact of transurethral procedures for benign prostate hyperplasia on male sexual function: A meta-analysis. J. Androl..

[76] Liu Y., Cheng Y., Zhuo L., Liu K., Xiao C., Zhao R., Lu J., Ma L. (2020). Impact on Sexual Function of Endoscopic Enucleation vs Transurethral Resection of the Prostate for Lower Urinary Tract Symptoms Due to Benign Prostatic Hyperplasia: A Systematic Review and Meta-Analysis. J. Endourol..

[77] Cacciamani G.E., Cuhna F., Tafuri A., Shakir A., Cocci A., Gill K., Gomez Rivas J., Dourado A., Veneziano D., Okhunov Z. (2019). Anterograde ejaculation preservation after endoscopic treatments in patients with bladder outlet obstruction: Systematic review and pooled-analysis of randomized clinical trials. Minerva Urol. Nefrol..

[78] Li Z., Chen P., Wang J., Mao Q., Xiang H., Wang X., Wang X., Zhang X. (2016). The impact of surgical treatments for lower urinary tract symptoms/benign prostatic hyperplasia on male erectile function: A systematic review and network meta-analysis. Medicine.

[79] Kim M., Song S.H., Ku J.H., Kim H.J., Paick J.S. (2015). Pilot study of the clinical efficacy of ejaculatory hood sparing technique for ejaculation preservation in Holmium laser enucleation of the prostate. Int. J. Impot. Res..

[80] Li M., Qiu J., Hou Q., Wang D., Huang W., Hu C., Li K., Gao X. (2015). Endoscopic enucleation versus open prostatectomy for treating large benign prostatic hyperplasia: A meta-analysis of randomized controlled trials. PLoS ONE.

[81] Lin Y., Wu X., Xu A., Ren R., Zhou X., Wen Y., Zou Y., Gong M., Liu C., Su Z. (2016). Transurethral enucleation of the prostate versus transvesical open prostatectomy for large benign prostatic hyperplasia: A systematic review and meta-analysis of randomized controlled trials. World J. Urol..

[82] Shoji S., Hanada I., Otaki T., Ogawa T., Yamada K., Uchida T., Higure T., Kawakami M., Kim H., Nitta M. (2020). Functional outcomes of transurethral thulium laser enucleation versus bipolar transurethral resection for benign prostatic hyperplasia over a period of 12 months: A prospective randomized study. Int. J. Urol..

[83] DeLay K.J., Nutt M., McVary K.T. (2016). Ejaculatory dysfunction in the treatment of lower urinary tract symptoms. Transl. Androl. Urol..

[84] Bachmann A., Tubaro A., Barber N., d’Ancona F., Muir G., Witzsch U., Grimm M.O., Benejam J., Stolzenburg J.U., Riddick A. (2014). 180-W XPS GreenLight laser vaporisation versus transurethral resection of the prostate for the treatment of benign prostatic obstruction: 6-month safety and efficacy results of a European Multicentre Randomised Trial--the GOLIATH study. Eur. Urol..

[85] Thomas J.A., Tubaro A., Barber N., d’Ancona F., Muir G., Witzsch U., Grimm M.O., Benejam J., Stolzenburg J.U., Riddick A. (2016). A Multicenter Randomized Noninferiority Trial Comparing GreenLight-XPS Laser Vaporization of the Prostate and Transurethral Resection of the Prostate for the Treatment of Benign Prostatic Obstruction: Two-yr Outcomes of the GOLIATH Study. Eur. Urol..

[86] Elshal A.M., Elmansy H.M., Elkoushy M.A., Elhilali M.M. (2012). Male sexual function outcome after three laser prostate surgical techniques: A single center perspective. Urology.

[87] Kavoussi P.K., Hermans M.R. (2008). Maintenance of erectile function after photoselective vaporization of the prostate for obstructive benign prostatic hyperplasia. J. Sex Med..

[88] Bruyere F. (2011). The relationship between photoselective vaporization of the prostate and sexual function. Curr. Urol. Rep..

[89] Alivizatos G., Skolarikos A., Chalikopoulos D., Papachristou C., Sopilidis O., Dellis A., Kastriotis I., Deliveliotis C. (2008). Transurethral photoselective vaporization versus transvesical open enucleation for prostatic adenomas >80 mL: 12-mo results of a randomized prospective study. Eur. Urol..

[90] Bouchier-Hayes D.M., Anderson P., Van Appledorn S., Bugeja P., Costello A.J. (2006). KTP laser versus transurethral resection: Early results of a randomized trial. J. Endourol..

[91] Bruyere F., Puichaud A., Pereira H., Faivre d’Arcier B., Rouanet A., Floc’h A.P., Bodin T., Brichart N. (2010). Influence of photoselective vaporization of the prostate on sexual function: Results of a prospective analysis of 149 patients with long-term follow-up. Eur. Urol..

[92] Razzaghi M.R., Mazloomfard M.M., Mokhtarpour H., Moeini A. (2014). Diode laser (980 nm) vaporization in comparison with transurethral resection of the prostate for benign prostatic hyperplasia: Randomized clinical trial with 2-year follow-up. Urology.

[93] Roehrborn C.G., Gange S.N., Gittelman M.C., Goldberg K.A., Patel K., Shore N.D., Levin R.M., Rousseau M., Beahrs J.R., Kaminetsky J. (2017). Convective Thermal Therapy: Durable 2-Year Results of Randomized Controlled and Prospective Crossover Studies for Treatment of Lower Urinary Tract Symptoms Due to Benign Prostatic Hyperplasia. J. Urol..

[94] McVary K.T., Rogers T., Roehrborn C.G. (2019). Rezum Water Vapor Thermal Therapy for Lower Urinary Tract Symptoms Associated With Benign Prostatic Hyperplasia: 4-Year Results From Randomized Controlled Study. Urology.

[95] McVary K.T., Gange S.N., Gittelman M.C., Goldberg K.A., Patel K., Shore N.D., Levin R.M., Rousseau M., Beahrs J.R., Kaminetsky J. (2016). Erectile and Ejaculatory Function Preserved With Convective Water Vapor Energy Treatment of Lower Urinary Tract Symptoms Secondary to Benign Prostatic Hyperplasia: Randomized Controlled Study. J. Sex Med..

[96] McVary K.T., Gange S.N., Gittelman M.C., Goldberg K.A., Patel K., Shore N.D., Levin R.M., Rousseau M., Beahrs J.R., Kaminetsky J. (2016). Minimally Invasive Prostate Convective Water Vapor Energy Ablation: A Multicenter, Randomized, Controlled Study for the Treatment of Lower Urinary Tract Symptoms Secondary to Benign Prostatic Hyperplasia. J. Urol..

[97] McVary K.T., Roehrborn C.G. (2018). Three-Year Outcomes of the Prospective, Randomized Controlled Rezum System Study: Convective Radiofrequency Thermal Therapy for Treatment of Lower Urinary Tract Symptoms Due to Benign Prostatic Hyperplasia. Urology.

[98] Darson M.F., Alexander E.E., Schiffman Z.J., Lewitton M., Light R.A., Sutton M.A., Delgado-Rodriguez C., Gonzalez R.R. (2017). Procedural techniques and multicenter postmarket experience using minimally invasive convective radiofrequency thermal therapy with Rezum system for treatment of lower urinary tract symptoms due to benign prostatic hyperplasia. Res. Rep. Urol..

[99] Dixon C.M., Cedano E.R., Pacik D., Vit V., Varga G., Wagrell L., Larson T.R., Mynderse L.A. (2016). Two-year results after convective radiofrequency water vapor thermal therapy of symptomatic benign prostatic hyperplasia. Res. Rep. Urol..

[100] Mollengarden D., Goldberg K., Wong D., Roehrborn C. (2018). Convective radiofrequency water vapor thermal therapy for benign prostatic hyperplasia: A single office experience. Prostate Cancer Prostatic Dis..

[101] Yalcin S., Tunc L. (2020). Indications, techniques, and role of new minimally invasive benign prostate hyperplasia surgical options. Turk J. Urol..

[102] Das A.K., Leong J.Y., Roehrborn C.G. (2019). Office-based therapies for benign prostatic hyperplasia: A review and update. Can. J. Urol..

[103] Spinos T., Katafigiotis I., Leotsakos I., Grivas N., Zabaftis C., Ermidis D., Sfoungaristos S., Karavitakis M. (2023). Rezum water vapor therapy for the treatment of patients with urinary retention and permanent catheter dependence secondary to benign prostate hyperplasia: A systematic review of the literature. World J. Urol..

[104] MacRae C., Gilling P. (2016). How I do it: Aquablation of the prostate using the AQUABEAM system. Can. J. Urol..

[105] Gilling P., Barber N., Bidair M., Anderson P., Sutton M., Aho T., Kramolowsky E., Thomas A., Cowan B., Kaufman R.P. (2018). WATER: A Double-Blind, Randomized, Controlled Trial of Aquablation((R)) vs Transurethral Resection of the Prostate in Benign Prostatic Hyperplasia. J. Urol..

[106] Kasivisvanathan V., Hussain M. (2018). Aquablation versus transurethral resection of the prostate: 1 year United States—Cohort outcomes. Can. J. Urol..

[107] Gilling P.J., Barber N., Bidair M., Anderson P., Sutton M., Aho T., Kramolowsky E., Thomas A., Cowan B., Roehrborn C. (2019). Randomized Controlled Trial of Aquablation versus Transurethral Resection of the Prostate in Benign Prostatic Hyperplasia: One-year Outcomes. Urology.

[108] Gilling P., Reuther R., Kahokehr A., Fraundorfer M. (2016). Aquablation—image-guided robot-assisted waterjet ablation of the prostate: Initial clinical experience. BJU Int..

[109] Gilling P., Anderson P., Tan A. (2017). Aquablation of the Prostate for Symptomatic Benign Prostatic Hyperplasia: 1-Year Results. J. Urol..

[110] Nguyen D.D., Barber N., Bidair M., Gilling P., Anderson P., Zorn K.C., Badlani G., Humphreys M., Kaplan S., Kaufman R. (2020). Waterjet Ablation Therapy for Endoscopic Resection of prostate tissue trial (WATER) vs WATER II: Comparing Aquablation therapy for benign prostatic hyperplasia in 30–80 and 80–150 mL prostates. BJU Int..

[111] Bhojani N., Bidair M., Zorn K.C., Trainer A., Arther A., Kramolowsky E., Doumanian L., Elterman D., Kaufman R.P., Lingeman J. (2019). Aquablation for Benign Prostatic Hyperplasia in Large Prostates (80–150 cc): 1-Year Results. Urology.

[112] Abt D., Hechelhammer L., Mullhaupt G., Markart S., Gusewell S., Kessler T.M., Schmid H.P., Engeler D.S., Mordasini L. (2018). Comparison of prostatic artery embolisation (PAE) versus transurethral resection of the prostate (TURP) for benign prostatic hyperplasia: Randomised, open label, non-inferiority trial. BMJ.

[113] Zhang J.L., Wang M.Q., Shen Y.G., Ye H.Y., Yuan K., Xin H.N., Zhang H.T., Fu J.X., Yan J.Y., Wang Y. (2019). Effectiveness of Contrast-enhanced MR Angiography for Visualization of the Prostatic Artery prior to Prostatic Arterial Embolization. Radiology.

[114] Pisco J.M., Bilhim T., Costa N.V., Torres D., Pisco J., Pinheiro L.C., Oliveira A.G. (2020). Randomised Clinical Trial of Prostatic Artery Embolisation Versus a Sham Procedure for Benign Prostatic Hyperplasia. Eur. Urol..

[115] Zumstein V., Betschart P., Vetterlein M.W., Kluth L.A., Hechelhammer L., Mordasini L., Engeler D.S., Kessler T.M., Schmid H.P., Abt D. (2019). Prostatic Artery Embolization versus Standard Surgical Treatment for Lower Urinary Tract Symptoms Secondary to Benign Prostatic Hyperplasia: A Systematic Review and Meta-analysis. Eur. Urol. Focus.

[116] Bagla S., Smirniotopoulos J.B., Orlando J.C., van Breda A., Vadlamudi V. (2015). Comparative Analysis of Prostate Volume as a Predictor of Outcome in Prostate Artery Embolization. J. Vasc. Interv. Radiol..

[117] Wang M., Guo L., Duan F., Yuan K., Zhang G., Li K., Yan J., Wang Y., Kang H., Wang Z. (2015). Prostatic arterial embolization for the treatment of lower urinary tract symptoms as a result of large benign prostatic hyperplasia: A prospective single-center investigation. Int. J. Urol..

[118] Pisco J., Bilhim T., Pinheiro L.C., Fernandes L., Pereira J., Costa N.V., Duarte M., Oliveira A.G. (2016). Prostate Embolization as an Alternative to Open Surgery in Patients with Large Prostate and Moderate to Severe Lower Urinary Tract Symptoms. J. Vasc. Interv. Radiol..

[119] Wang M., Guo L., Duan F., Yuan K., Zhang G., Li K., Yan J., Wang Y., Kang H. (2016). Prostatic arterial embolization for the treatment of lower urinary tract symptoms caused by benign prostatic hyperplasia: A comparative study of medium- and large-volume prostates. BJU Int..

[120] Wang X.Y., Zong H.T., Zhang Y. (2016). Efficacy and safety of prostate artery embolization on lower urinary tract symptoms related to benign prostatic hyperplasia: A systematic review and meta-analysis. Clin. Interv. Aging.

[121] Ray A.F., Powell J., Speakman M.J., Longford N.T., DasGupta R., Bryant T., Modi S., Dyer J., Harris M., Carolan-Rees G. (2018). Efficacy and safety of prostate artery embolization for benign prostatic hyperplasia: An observational study and propensity-matched comparison with transurethral resection of the prostate (the UK-ROPE study). BJU Int..

[122] Moreira A.M., de Assis A.M., Carnevale F.C., Antunes A.A., Srougi M., Cerri G.G. (2017). A Review of Adverse Events Related to Prostatic Artery Embolization for Treatment of Bladder Outlet Obstruction Due to BPH. Cardiovasc. Intervent. Radiol..

[123] Abt D., Mullhaupt G., Hechelhammer L., Markart S., Gusewell S., Schmid H.P., Mordasini L., Engeler D.S. (2021). Prostatic Artery Embolisation Versus Transurethral Resection of the Prostate for Benign Prostatic Hyperplasia: 2-yr Outcomes of a Randomised, Open-label, Single-centre Trial. Eur. Urol..

[124] Naidu S.G., Narayanan H., Saini G., Segaran N., Alzubaidi S.J., Patel I.J., Oklu R. (2021). Prostate Artery Embolization-Review of Indications, Patient Selection, Techniques and Results. J. Clin. Med..

[125] Chin P.T., Bolton D.M., Jack G., Rashid P., Thavaseelan J., Yu R.J., Roehrborn C.G., Woo H.H. (2012). Prostatic urethral lift: Two-year results after treatment for lower urinary tract symptoms secondary to benign prostatic hyperplasia. Urology.

[126] McNicholas T.A., Woo H.H., Chin P.T., Bolton D., Fernandez Arjona M., Sievert K.D., Schoenthaler M., Wetterauer U., Vrijhof E.J., Gange S. (2013). Minimally invasive prostatic urethral lift: Surgical technique and multinational experience. Eur. Urol..

[127] Roehrborn C.G., Gange S.N., Shore N.D., Giddens J.L., Bolton D.M., Cowan B.E., Brown B.T., McVary K.T., Te A.E., Gholami S.S. (2013). The prostatic urethral lift for the treatment of lower urinary tract symptoms associated with prostate enlargement due to benign prostatic hyperplasia: The L.I.F.T. Study. J. Urol..

[128] Roehrborn C.G., Barkin J., Gange S.N., Shore N.D., Giddens J.L., Bolton D.M., Cowan B.E., Cantwell A.L., McVary K.T., Te A.E. (2017). Five year results of the prospective randomized controlled prostatic urethral L.I.F.T. study. Can. J. Urol..

[129] McVary K.T., Gange S.N., Shore N.D., Bolton D.M., Cowan B.E., Brown B.T., Te A.E., Chin P.T., Rukstalis D.B., Roehrborn C.G. (2014). Treatment of LUTS secondary to BPH while preserving sexual function: Randomized controlled study of prostatic urethral lift. J. Sex Med..

[130] Sonksen J., Barber N.J., Speakman M.J., Berges R., Wetterauer U., Greene D., Sievert K.D., Chapple C.R., Montorsi F., Patterson J.M. (2015). Prospective, randomized, multinational study of prostatic urethral lift versus transurethral resection of the prostate: 12-month results from the BPH6 study. Eur. Urol..

[131] Xiang P., Wang M., Guan D., Liu D., Wang Y., Hao Y., Li S., Liu Y., Ping H. (2020). A Systematic Review and Meta-analysis of Prostatic Urethral Lift for Male Lower Urinary Tract Symptoms Secondary to Benign Prostatic Hyperplasia. Eur. Urol. Open Sci..

[132] Perera M., Roberts M.J., Doi S.A., Bolton D. (2015). Prostatic urethral lift improves urinary symptoms and flow while preserving sexual function for men with benign prostatic hyperplasia: A systematic review and meta-analysis. Eur. Urol..

[133] Jung J.H., Reddy B., McCutcheon K.A., Borofsky M., Narayan V., Kim M.H., Dahm P. (2019). Prostatic urethral lift for the treatment of lower urinary tract symptoms in men with benign prostatic hyperplasia. Cochrane Database Syst. Rev..

[134] Sievert K.D., Schonthaler M., Berges R., Toomey P., Drager D., Herlemann A., Miller F., Wetterauer U., Volkmer B., Gratzke C. (2019). Minimally invasive prostatic urethral lift (PUL) efficacious in TURP candidates: A multicenter German evaluation after 2 years. World J. Urol..

[135] Dean N.S., Assmus M.A., Lee M.S., Guo J.N., Krambeck A.E. (2023). Benign prostatic hyperplasia surgical re-treatment after prostatic urethral lift A narrative review. Can. Urol. Assoc. J..

[136] Long J.D., Smith S.R., Members of the Cochrane Nursing C. (2021). Prostatic urethral lift for the treatment of lower urinary tract symptoms in men with benign prostatic hyperplasia: A Cochrane review summary. Int. J. Nurs. Stud..

[137] Magistro G., Stief C.G., Gratzke C. (2015). New intraprostatic injectables and prostatic urethral lift for male LUTS. Nat. Rev. Urol..

[138] Elhilali M.M., Pommerville P., Yocum R.C., Merchant R., Roehrborn C.G., Denmeade S.R. (2013). Prospective, randomized, double-blind, vehicle controlled, multicenter phase IIb clinical trial of the pore forming protein PRX302 for targeted treatment of symptomatic benign prostatic hyperplasia. J. Urol..

[139] El-Dakhakhny A.S., Gharib T., Issam A., El-Karamany T.M. (2019). Transperineal intraprostatic injection of botulinum neurotoxin A vs transurethral resection of prostate for management of lower urinary tract symptoms secondary to benign prostate hyperplasia: A prospective randomised study. Arab. J. Urol..

[140] Porpiglia F., Fiori C., Bertolo R., Giordano A., Checcucci E., Garrou D., Cattaneo G., De Luca S., Amparore D. (2018). 3-Year follow-up of temporary implantable nitinol device implantation for the treatment of benign prostatic obstruction. BJU Int..

[141] Pham H., Sharma P. (2018). Emerging, newly-approved treatments for lower urinary tract symptoms secondary to benign prostatic hypertrophy. Can. J. Urol..

[142] Chughtai B., Elterman D., Shore N., Gittleman M., Motola J., Pike S., Hermann C., Terrens W., Kohan A., Gonzalez R.R. (2021). The iTind Temporarily Implanted Nitinol Device for the Treatment of Lower Urinary Tract Symptoms Secondary to Benign Prostatic Hyperplasia: A Multicenter, Randomized, Controlled Trial. Urology.

[143] Porpiglia F., Fiori C., Amparore D., Kadner G., Manit A., Valerio M., Nicolaas L., Ho B.S.H., Alonso S., Schulman C. (2019). Second-generation of temporary implantable nitinol device for the relief of lower urinary tract symptoms due to benign prostatic hyperplasia: Results of a prospective, multicentre study at 1 year of follow-up. BJU Int..

[144] Li M.K., Garcia L., Patron N., Moh L.C., Sundram M., Leungwattanakij S., Pripatnanont C., Cheng C., Chi-Wai M., Loi-Cheong N. (2008). An Asian multinational prospective observational registry of patients with benign prostatic hyperplasia, with a focus on comorbidities, lower urinary tract symptoms and sexual function. BJU Int..

[145] Chung A., Woo H.H. (2016). Preservation of sexual function when relieving benign prostatic obstruction surgically: Can a trade-off be considered?. Curr. Opin. Urol..

[146] Talab S.S., Santiago-Lastra Y.A., Bachmann A., Choi B.B., Muir G.H., Woo H.H., Tabatabaei S. (2013). V403 the impact of ejaculation-preserving photo-selective vaporization of the prostate (ep-pvp) on lower urinary tract symptoms and ejaculatory function: Results of a multicenter study. J. Urol..

[147] Alloussi S.H., Lang C., Eichel R., Alloussi S. (2014). Ejaculation-preserving transurethral resection of prostate and bladder neck: Short- and long-term results of a new innovative resection technique. J. Endourol..

[148] Wang P., Xia D., Ye S., Kong D., Qin J., Jing T., Mao Y., Meng H., Wang S. (2018). Robotic-assisted Urethra-sparing Simple Prostatectomy Via an Extraperitoneal Approach. Urology.

[149] Bowen D.K., Butcher M.J., Botchway A., McVary K.T. (2015). Counseling on sexual side effects from TURP. Can. J. Urol..

[150] Albaugh J.A., Sufrin N., Lapin B.R., Petkewicz J., Tenfelde S. (2017). Life after prostate cancer treatment: A mixed methods study of the experiences of men with sexual dysfunction and their partners. BMC Urol..

[151] Patel R.M., Bariol S. (2019). National trends in surgical therapy for benign prostatic hyperplasia in Australia. ANZ J. Surg..

[152] Malaeb B.S., Yu X., McBean A.M., Elliott S.P. (2012). National trends in surgical therapy for benign prostatic hyperplasia in the United States (2000–2008). Urology.

[153] Carmignani L., Bozzini G., Macchi A., Maruccia S., Picozzi S., Casellato S. (2015). Sexual outcome of patients undergoing thulium laser enucleation of the prostate for benign prostatic hyperplasia. Asian J. Androl..

[154] Al Rawashdah S.F., Pastore A.L., Velotti G., Fuschi A., Capone L., Suraci P.P., Martoccia A., Saltarelli A., Minucci S., Falsaperla M. (2020). Sexual and functional outcomes of prostate artery embolisation: A prospective long-term follow-up, large cohort study. Int. J. Clin. Pract..

[155] Kim S.H., Yang H.K., Lee H.E., Paick J.S., Oh S.J. (2014). HoLEP does not affect the overall sexual function of BPH patients: A prospective study. Asian J. Androl..

[156] Sato R., Sano A., Watanabe K., Matsushita Y., Watanabe H., Tamura K., Motoyama D., Sugiyama T., Otsuka A., Miyake H. (2022). Effects of Changes in Erectile Function After Holmium Laser Enucleation of the Prostate on Postoperative Outcomes in Patients With Benign Prostatic Hyperplasia. In Vivo.

[157] Jeong M.S., Ha S.B., Lee C.J., Cho M.C., Kim S.W., Paick J.S. (2012). Serial Changes in Sexual Function Following Holmium Laser Enucleation of the Prostate: A Short-term Follow-up Study. Korean J. Urol..

[158] Bozzini G., Berti L., Maltagliati M., Besana U., Calori A., Muller A., Sighinolfi M.C., Micali S., Pastore A.L., Ledezma R. (2021). Ejaculation-sparing thulium laser enucleation of the prostate (ES-ThuLEP): Outcomes on a large cohort. World J. Urol..

[159] Fogaing C., Alsulihem A., Campeau L., Corcos J. (2021). Is Early Surgical Treatment for Benign Prostatic Hyperplasia Preferable to Prolonged Medical Therapy: Pros and Cons. Medicina.

[160] Basson R., Rees P., Wang R., Montejo A.L., Incrocci L. (2010). Sexual function in chronic illness. J. Sex Med..

